# 348. Cell-free plasma next-generation sequencing assists in the evaluation of invasive mold infection in patients with COVID-19: a case series

**DOI:** 10.1093/ofid/ofac492.426

**Published:** 2022-12-15

**Authors:** Joshua David, Bharadhwaj Kolipakkam, Megan M Morales, Nicole C Vissichelli

**Affiliations:** Virginia Commonwealth University School of Medicine, Richmond, Virginia; Virginia Commonwealth University Health System, Richmond, Virginia; Virginia Commonwealth University Health System, Richmond, Virginia; Virginia Commonwealth University Health System, Richmond, Virginia

## Abstract

**Background:**

Distinguishing COVID-19 Associated Pulmonary Aspergillosis (CAPA) and invasive mold infections (IMIs) from other causes of secondary pneumonia in COVID-19 can be challenging. 1,3-β-D-Glucan and galactomannan are commonly utilized biomarkers for the workup of IMIs but are limited by a lack of specificity and sensitivity respectively. Cell-free plasma next-generation sequencing (cfNGS) is a promising non-invasive approach that can provide direct detection of pathogens in patient's serum. This study explored its potential role in the evaluation of secondary pneumonia in patients with COVID-19.

**Methods:**

We performed a retrospective single-center observational study from March 2020 to December 2021 at Virginia Commonwealth University Medical Center, a 811-bed tertiary care center, to evaluate patients with laboratory confirmed SARS-CoV-2 virus infection who underwent cfNGS for the evaluation of CAPA. CfNGS (Karius, Inc., Redwood City, CA) was performed at the discretion of the clinical provider and we evaluated the test indication, patient history, clinical impact, correlation with serum biomarkers, and 30 day all-cause mortality.

**Results:**

Thirteen patients were evaluated and none had *Aspergillus* species detected. One patient had *Pneumocystis jirovecii* on cfNGS. There was a 76.9% (10/13) concordance rate with patients’ serum fungal biomarkers. CfNGS also detected concomitant organisms in 53.8% (7/13) of our cohort. These data assisted in changes of clinical management for 84.6% (11/13) of patients and lead to the change in antifungal usage in 69.2% (9/13).

**Figure:**
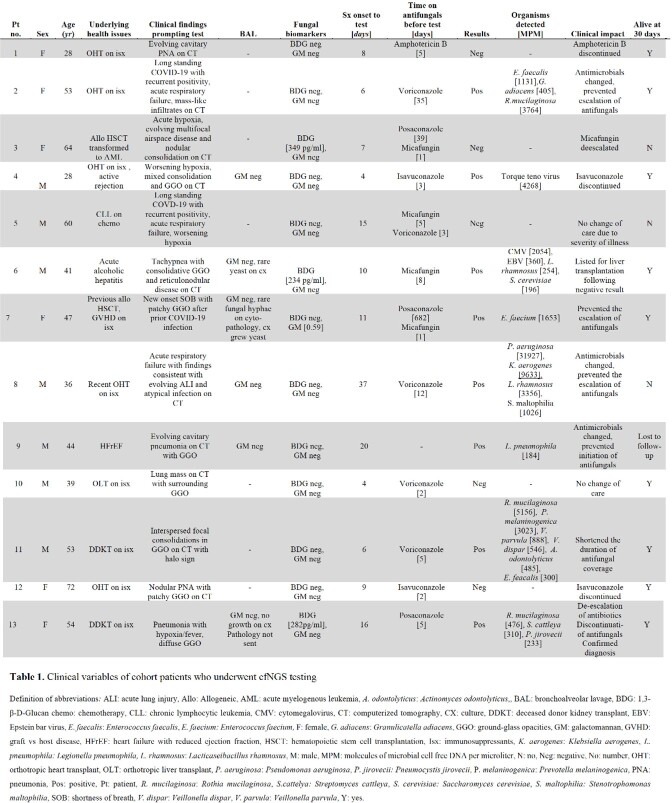

**Conclusion:**

In this study, both negative and positive cfNGS test results assisted in important clinical decision making. cfNGS may have a role in the evaluation of CAPA or other IMIs in patients with COVID-19.

**Disclosures:**

**Megan M. Morales, MD**, Karius: Paid speaker.

